# Impact of COVID-19 Pandemic on the Use of Antidepressant and Antianxiety Pharmaceuticals as Well as Sick Leave in Poland

**DOI:** 10.3390/ijerph19042135

**Published:** 2022-02-14

**Authors:** Dominika Krupa, Marcin Czech, Jarosław Pinkas, Anna Mosiołek

**Affiliations:** 1 Faculty of Management, University of Warsaw, 02-678 Warsaw, Poland; 2 Department of Pharmacoeconomics, Institute of Mother and Child, 01-211 Warsaw, Poland; marcin.czech@imid.med.pl; 3 School of Public Health, Centre of Postgraduate Medical Education, 01-826 Warsaw, Poland; jaroslaw.pinkas@cmkp.edu.pl; 4 Department of Psychiatry, Faculty of Health Sciences, Medical University of Warsaw, 05-802 Pruszków, Poland; manitka@tlen.pl

**Keywords:** COVID-19, mental health, antidepressants, antianxiety, pharmaceuticals, sick leave, depression, anxiety, Poland

## Abstract

The COVID-19 pandemic caused a major upheaval to the lives of people and placed a strain on societal mental health. The aim of this research is to estimate the impact of the pandemic on the mental condition of the Polish population measured through the consumption of relevant medication and medical leave of absence from the workplace. Methods: We analyzed national-level data on the consumption of pharmaceuticals used in clinical practice in Poland in the treatment of depression and anxiety alongside medical absence in the workplace using the Interrupted Time Series model to estimate the significance of the pandemic. Results: We found no significant change regarding the consumption of pharmaceuticals with the development of the pandemic. Conversely, medical leaves of absence for psychiatric reasons increased significantly with the onset of COVID-19. The influence was strongest in the diagnosis of anxiety or reaction to severe stress and weakest in recurrent depression. Conclusion: The pandemic had a significant influence on the ability to work for psychiatric patients in Poland but did not change pharmaceutical use. Physicians should consider the mental health of patients impacted by the anti-epidemic measures. Further study is needed to fully understand the long-term impact of the pandemic on mental health in Poland.

## 1. Introduction

The onset of the global pandemic of COVID-19 caused by the novel coronavirus SARS-CoV-2 has caused a major disruption to everyday lives of people everywhere. The first cases were recorded in China in December 2019 and, by March 2020, had spread to most countries around the world, including Poland [1]. A state of epidemic was introduced in Poland on 20 March 2020 and, as of January 2022, was ongoing. Various measures of mobility restrictions and isolation were imposed to prevent exponential spread of the potentially deadly disease [2].

Periods of crisis and uncertainty, such as a global epidemic, are recognized as times of increased incidence of mental disorders in the population [3,4,5]. Specifically, a connection between mental illness and infectious disease outbreaks, including COVID-19, has also been described in literature [6]. For this reason, the importance of mental health resources for patients in such times has been recognized by researchers [7] as well as the World Health Organization [8].

Nevertheless, there is no official guideline or verification of the effectiveness or availability of mental health support, causing heterogeneity in patient experiences when it comes to psychiatric support between countries. Moreover, the scale of the impact of the pandemic on the mental health of societies in different geographies remains unknown. While some studies describing the impact of the pandemic on specific groups, including healthcare workers [9], the elderly [10], children [11] or new mothers [12] do emerge, their targeted approach limits the generalizability of the conclusions.

Until large-scale primary studies explicitly evaluating the mental health of populations in the pandemic are published, the question of the scale of the unmet need in psychiatric and psychological state of the population remains open and a burden to policy makers, employers, families and patients. In the meantime, secondary data is a viable source of knowledge that can guide research and decision makers.

Poland is the sixth largest country in the EU, with a population of almost 38 million people. The single payer public healthcare system is one of the lowest funded in the European Union, with 6.5% GDP spent on healthcare, compared to an EU average of 9.9% [13]. Psychiatry specifically is a specific of concern, since according to data from Eurostat, there are only nine psychiatrists for 100,000 inhabitants in Poland [14], a penultimate result in the EU, with only Bulgaria having less.

At the same time, there were 27 psychiatrists in Germany, 18 in Estonia and 15 in Hungary. The relatively low availability of specialists in psychiatry indicates that, in the extraordinary circumstances of a global pandemic, Polish patients may face difficulties in obtaining access to treatment, thus worsening their wellbeing. In this paper, we aim to estimate the impact of COVID-19 on the mental health of the Polish population.

## 2. Materials and Methods

An analysis of two types of data was conducted. First, we looked at the consumption of antidepressants and antianxiety medication in the population. Secondly, we analyzed the information on the frequency and duration of sick leave due to psychiatric diagnosis.

For the consumption of medication, we analyzed data from IQVIA Pharmascope database on prescription only (Rx) purchases done by patients in community pharmacies. IQVIA Pharmascope is a national-level database containing information on all products purchased in community pharmacies across Poland. Available information for pharmaceuticals includes: pack details (active ingredient, pack size, dose, brand name, manufacturer, EPhMRA Anatomic Therapeutical Classification [15] (ATC) levels 1 through 4), pharmacy identifier, reimbursement status, date of purchase (by month), price of purchase and purchased volume.

We analyzed monthly data from January 2018 until October 2021 for drugs from classes N06A4 SSRI Antidepressants, N06A5 SNRI Antidepressants, N06A9 All Other Antidepressants and one molecule from the N05C0 Tranquillizers class (buspirone). Pharmaceutical consumption data was analyzed in both value and volume, on SKU (Stock Keeping Unit) level as well as the size of therapeutic doses purchased measured through Daily Defined Dose (DDD) for each molecule specified by the World Health Organization [16].

We looked at aggregated trends as well as analysis within the medication subgroups. The database does not contain information on patient characteristics, diagnosis, or the specialty of the physician issuing the prescription, therefore the presented figures will include treatment for multiple disorders as well as potential off-label use of the medication. To limit the effect of potential use in other conditions, verification of molecules used in clinical practice of depression and anxiety management in Poland was conducted by a clinician.

The impact of depression and anxiety on the workforce was analyzed on data on the frequency and duration of medical leave due to a diagnosis of psychiatric condition collected by the Social Insurance Institution of Poland (pol. *Zakład Ubezpieczeń Społecznych*, ZUS). ZUS is a state organizational unit in Poland, whose responsibilities include establishing entitlements to social transfers, collections of social security premiums (retirement, disability, sickness and healthcare) and payment of the benefits, among others [17].

All leaves of absence from the workplace, both short and long-term, must be reported to ZUS. Additionally, justification documentation for the absence for medical reasons must be issued by a physician with the authorization of ZUS and must include information on the diagnosis that is the direct cause of inability to work. For this research, we procured data on medical absence due to selected ICD10 diagnoses: F32—depressive episode; F33—recurrent depressive disorder; F41—other anxiety disorder; and F43—reaction to severe stress and adjustment disorders; spanning the period of January 2018 to April 2021. The scope of the data included the count of patients and average duration of sick leave.

Both types of collected data included a period before the onset of the pandemic, and several months of its duration in Poland. To compliment and contextualize the two primary datasets, additional sources were used. Data on the population of patients dealing with depression and/or anxiety in Poland were sourced from the National Health Fund publications and from the results of a large epidemiological study on mental illness in Poland—the EZOP project [18]. Data on the development of the pandemic in Poland for incidence, COVID and non-COVID-related mortality, as well as restriction stringency was sourced from OurWorldInData [1], an open-source database updated daily. For this paper, daily data for the period 1 January 2020 until 1 December 2021 was analyzed.

Interrupted Time Series analysis was used to validate impact of the onset of COVID pandemic on pharmaceutical consumption and number of issued sick leave notes. To estimate the significance of COVID-19 on the analyzed series, we used two synthetic variables—a dummy for the period of the duration of the pandemic (from March 2020 to October2021) and a linear trend in the same period to capture slope change.

Statistical analyses were conducted in the open-source software R, version x64 4.1.2 [19], using the RStudio interface. Data visualizations were done in MS Excel version 2102 (Microsoft Corporation, Redmond, WA, USA).

## 3. Results

The estimated prevalence of depression in adults in Poland was estimated at 3.17% in the Global Burden of Disease Study [20] for the general population, and at 3.85% in the results of the latest nation-wide epidemiological study EZOP II for adults [21]. This corresponds to a population estimate between 1.2–1.45 million. Simultaneously, the lifetime prevalence of anxiety attacks is estimated at 7% in the EZOP II study, equivalent to 1.3% annually. The authors estimate that there are around 2.2 million people who have suffered from anxiety in Poland. Data from the NFZ report that reimbursement for antidepressants and antianxiety medication has been granted to 1.38 million people in 2020 [22].

### 3.1. Drug Consumption

The overall Rx market in pharmacies was worth 4.4 billion EUR [23]. The antidepressant and antianxiety medications that are the focus of this study constitute approximately 2.6% of the total Rx market value. According to data from IQVIA Pharmascope, annually, patients in Poland purchased more than 20 million packs of antidepressants and antianxiety medication valued at more than 114 million EUR. The monthly consumption of pharmaceuticals in this category in 2021 amounted to 2.3 million packs for the cumulative value of 12 million EUR.

**Figure 1:**
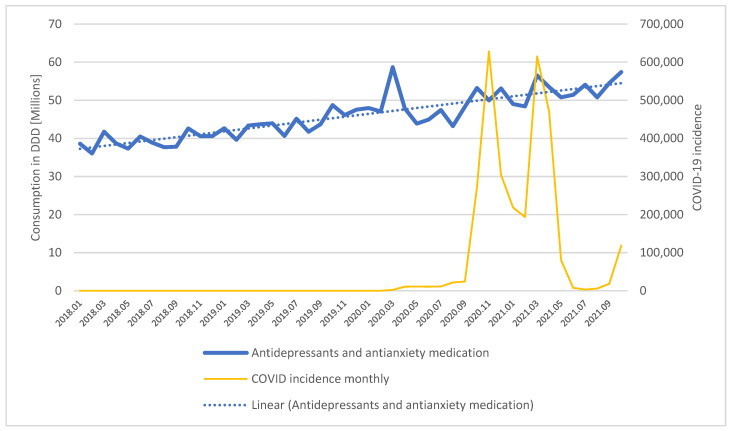
Consumption of all antidepressants and antianxiety medication in Poland in DDD, January 2018–October 2021.

The consumption of pharmaceuticals indicated for treating depression and anxiety has been increasing steadily in Poland over the last years. The monthly consumption of antidepressants and antianxiety medication between January 2018 and October 2021 increased from almost 40 million doses to almost 60 million. The increase follows a linear trend as presented in Figure 1.

A peak in purchases was recorded in March 2020 with the onset of pandemic in Poland, followed by slightly lower sales over the next 6 months and ultimately returned to the previous dynamics.

**Figure 2:**
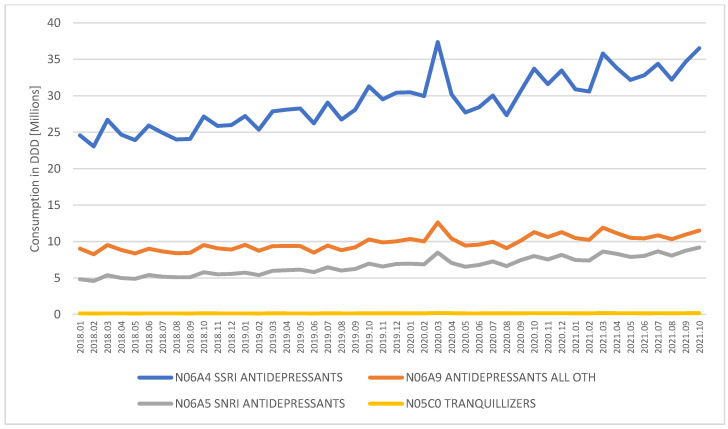
The consumption of antidepressants and antianxiety medication in Poland by ATC4 classification, January 2018–October 2021.

Development of specific categories is presented in Figure 2. The most popular products are Selective Serotonin Reuptake Inhibitors (SSRI), representing 64% of the entire volume, followed by antidepressants of other mechanisms with 21% of volume and Serotonin and Norepinephrine Reuptake Inhibitors (SNRI) with 14.5%. Buspirone molecule classified as tranquilizer covers 0.3% of volume. All categories of medication present similar development patterns in time, with SNRI growing at a slightly larger rate. Consistency in growth path is confirmed by high Pearson correlation factors along with significance levels at *p* < 0.01, as presented in Table 1.

Within the analyzed classes, the ten most popular molecules account for 91% of dose volume sales. Out of the ten, seven molecules are reimbursed and available in community pharmacies in Poland. For each molecule, the pack with the highest sales volume was selected to present the cost for the patient. The pack price for the most popular drugs ranges from 3.86 to 6.05 EUR. When purchased with reimbursement, the co-payment ranges from 0.75 to 2.44 EUR per pack. Table 2 presents the key characteristics of the most popular molecules in relevant therapy in Poland.

Between January 2018 and October 2021, the consumption of antidepressants and antianxiety medication in DDD grew by 48.8%. New products introduced into the market in that time accounted for 5.9% of growth. Previously available products pack volume increased by 27.7%. The average size of pack increased by 4.3%, while the average dose per tablet increased by 5.4%.

Since the data does not include information on the number of patients on therapy, it is not possible to precisely estimate the magnitude of the increase in patient population, since it is not known how many of the additional packs or new products were purchased by patients previously treated and how many were purchased as initiation within that period. However, an increase in pack size or in dose could have been purchased only by the same patient and, therefore, can be interpreted as therapy intensification. In the analyzed period, on average, the dose of medication increased by 10.0%, as patients purchased larger packs with higher doses per tablet.

Interrupted time series analysis of the drug consumption included three variables—underlying linear trend (denoted as Period), a dummy variable for the duration of the COVID pandemic (COVID fixed) and a linear trend from the onset of COVID-19 (COVID variable). The results of the regression are presented in Table 3. Neither COVID variable achieved statistical significance, confirming that no trend break occurred within the analyzed period, and the pandemic did not have a significant impact on the consumption of antidepressant and antianxiety medication in Poland in terms of volume.

### 3.2. Sick Leave

**Figure 3:**
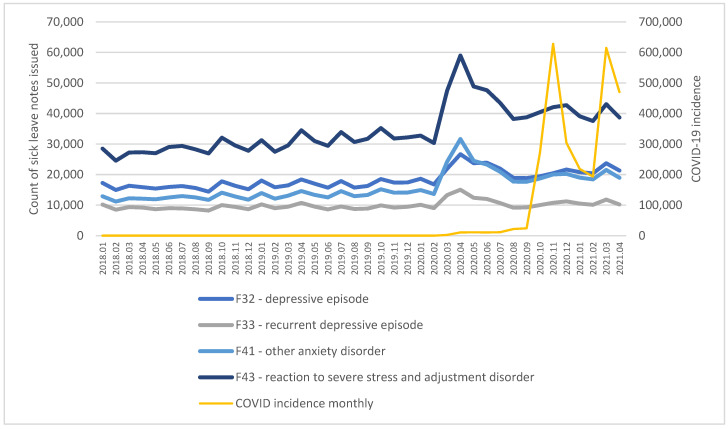
Count of sick leave notes issued in Poland, January 2018–April 2021.

Before the pandemic there were roughly 70,000 sick leave notes issued for the diagnoses related to depression and anxiety in Poland monthly. Between January 2018 and January 2020, the number has been increasing at a pace of 0.44% monthly. When the pandemic started in March 2020, the number of sick notes grew to 107,000 in March 2020 and reached a peak in April 2020 at 132,000. Dynamics by specific diagnoses are presented in Figure 3. The intensive period of increased sick leave issuance lasted for about six months until August 2020 and later stabilized. However, some lasting effects are visible, as the level of notes prescribed for F43 visibly did not return to levels from before the pandemic.

Interrupted time series analysis further confirmed the conclusion that COVID had a significant influence on the number of sick leave notes. Detailed results of the regression are presented in Table 4. Out of the four analyzed diagnoses, before the onset of the pandemic only F43 was characterized by a significant upward trend (*p* < 0.01). However, for each of the diagnoses, the COVID fixed dummy variable had a significant positive influence on the number of sick leave notes issued (*p* < 0.001). The strongest relative effect was observed for F41 diagnosis and the weakest for F33, recurrent depressive episodes. Linear trend associated with COVID was also significant for each diagnosis and was negative, indicating a decreasing impact of the pandemic over time.

## 4. Discussion

The data on pharmaceutical drug consumption and medical leaves of absence indicates that the impact of COVID-19 pandemic had mixed effects on patients suffering from depression or anxiety in Poland. On one hand, medical absence associated with diagnosis of anxiety or depression increased significantly with the onset of restrictions aimed at combatting the pandemic. This suggests either that patients observed worsening of their condition or that the population increased. On the other hand, pharmaceutical consumption growth does not mirror this behavior, having increased in the initial month only slightly before returning to the long-term dynamics.

On its own, the lack of significant increase in consumption associated with COVID-19 stands in contrary to data from the UK [24], where researchers found a significant surge in pharmaceutical use in the pandemic. In Germany [25], a decrease in the prescription of drugs for mental disorders was identified. Interestingly, similarly to this study, research from Portugal found no impact of the pandemic on the increasing prescribing trends of drugs of interest [26].

The hypothesis for the disparity could therefore be that patients suffering from depression and anxiety (in Poland) did not fare worse when faced with the new reality—or, alternatively, it indicates that a potentially large population of patients should be undergoing pharmacotherapy but are not, either due to a lack of awareness, unwillingness to try, poor compliance or limited access to psychiatric care, putting them at higher risk of adverse events associated with depression and anxiety. It is, however, also possible that the data used in this study does not accurately capture the impact of the pandemic on the consumption of prescription medication. This would indicate a potentially large population of patients at higher risk of adverse events associated with depression and anxiety [27].

The hypothesis of underdiagnosis of depression in Poland can be strengthened by information presented within the Global Burden of Disease study [20], where the depression and anxiety rates per 10,000 population in Poland in 2019 were estimated at 2.836 (14% lower than Central European countries (Countries in Central Europe as defined by the Institute of Health Metrics and Evaluation are: Albania, Bosnia and Herzegovina, Bulgaria, Croatia, Czech Republic, Hungary, Montenegro, North Macedonia, Poland, Romania, Serbia, Slovakia and Slovenia)) and 3.489 (6% lower than the region) respectively.

The argument for underdiagnosis of those mental conditions in Poland is stronger, since while, historically and socially, the countries have many similarities, Poland is one of the better developed economically, suggesting potentially better access to healthcare services. Globally, the use of antidepressants and antianxiety medication is growing, particularly in high income countries [28].

Between 2008 and 2019, the average consumption per day per 1000 inhabitants in high income countries measured by daily defined dose increased from 51.98 to 72.93, indicating a 40% increase and equivalent to an average compound growth of 3% per year [28]. According to the data obtained for this study, in January 2018, the DDD consumption for 1000 citizens per day was 32.94 doses; whereas, in October 2021, it was 49.01, representing a 49% increase over a little under three years, rather than 12 years.

This suggests that consumption of antidepressants and antianxiety medication in Poland vastly outpaces the developed world. Nevertheless, the levels remain below the average consumption in high income countries in 2008, showing the level of the gap still to be breached to improve the care of patients suffering from depression or anxiety in Poland.

Alternatively, the patients may not have had access to doctors who would be able to issue a prescription. This hypothesis is however disproven by the increase in sick leave notes, which must be issued by doctors. Therefore, it is possible that the patients who obtained medical leave privileges are not undergoing treatment for their mental health problems. This could explain some increase in sick leave notes issued, as it is possible that workers in unsteady employment were afraid of losing their job under the unpredictable circumstances brought around by COVID-19. The relatively low cost of pharmacotherapy, with monthly cost starting at 0.75 EUR per month of therapy, indicates that economic barriers are not the primary limiting factor for treatment.

Verification of those hypotheses warrants further study whenever new data becomes available, as they carry important implications to health policy.

This study has several limitations. As the pandemic was ongoing as this research was being published, it was impossible to assess the full impact of COVID-19 on mental health in Polish patients. Secondly, the data obtained enables analysis on a level of aggregation which may obstruct the detection of underlying granular trends. Thirdly, the low number of observations in the analyzed time series inflates the uncertainty in estimates by reducing the scope for more sophisticated numerical analysis. The monthly format of the data inhibits the use of more time-sensitive variables, such as governmental restrictions, whose impact on drug consumption or sick leave would need to be quantified using higher granularity observations.

## 5. Conclusions

Although the COVID-19 pandemic had a significant impact on the frequency and duration of sick leaves due to depression or anxiety in Poland, the consumption of pharmaceuticals remained largely unaffected and has been increasing at a consistent rate since 2018. Further study is needed to fully understand the reasons behind this and to determine whether the cause lies in consistently insufficient awareness of treatment in the population, restricted access to psychiatric care or alternative methods of coping with depression prevalent within the Polish population.

This study showed that the COVID-19 pandemic may have a significant impact on mental health. Physicians should pay attention to the mental health problems among patients who were affected by the anti-epidemic measures, such as lock-down and remote work. Moreover, public health programs are needed to address the growing burden of mental health illnesses.

## Figures and Tables

**Table 1:** Correlation matrix between drug classes in the period January 2018–October 2021.

	N06A4 SSRI	N06A9 OTHER	N06A5 SNRI	N05C0 TRANQUILLIZERS
N06A4 SSRI	1.00			
N06A9 OTHER	0.97 ***	1.00		
N06A5 SNRI	0.98 ***	0.92 ***	1.00	
N05C0 TRANQUILLIZERS	0.95 ***	0.96 ***	0.91 ***	1.00

Note: *** *p* < 0.01. N06A4 SSRI—Selective Serotonin Reuptake Inhibitors; N06A5 SNRI—Serotonin-Norepinephrine Reuptake Inhibitors; N06A9 OTHER—all other antidepressants not classified elsewhere.

**Table 2:** Most popular antidepressant and antianxiety medication in Poland, 2021.

Molecule	ATC4 Category	Reimbursement Status in Poland [September 2021]	DDD [mg]	Most Popular Pack	DDD Consumed 2021 (January–October), Millions	Share of DDD Consumption in 2021 (January–October), %	2021 Market Value (January–October), Million EUR	2021 Market Volume (January–October), Million Packs	Average Pack Price, EUR	Average Co-Payment to Most Popular Pack [September 2021], EUR
SERTRALINE	N06A4 SSRI ANTIDEPRESSANTS	Yes	50	50 mg 30 tabs	133.01	25%	13.73	3.56	3.86	0.92
ESCITALOPRAM	N06A4 SSRI ANTIDEPRESSANTS	No	10	10 mg 28 tabs	82.88	16%	13.59	2.76	4.91	n/a
VENLAFAXINE	N06A5 SNRI ANTIDEPRESSANTS	Yes	100	75 mg 28 caps, extended release	58.64	11%	13.24	2.34	5.67	1.67
FLUOXETINE	N06A4 SSRI ANTIDEPRESSANTS	Yes	20	20 mg 30 tabs	40.11	8%	6.33	1.33	4.76	1.78
CITALOPRAM	N06A4 SSRI ANTIDEPRESSANTS	No	20	20 mg 28 tabs	38.68	7%	5.95	1.28	4.64	n/a
PAROXETINE	N06A4 SSRI ANTIDEPRESSANTS	Yes	20	20 mg 30 tabs	36.73	7%	4.89	1.10	4.43	1.64
OPIPRAMOL	N06A9 ANTIDEPRESSANTS ALL OTH	No	150	50 mg 56 tabs	26.60	5%	11.19	1.89	5.93	n/a
TRAZODONE	N06A9 ANTIDEPRESSANTS ALL OTH	Yes	300	75 mg 30 tabs, extended release	26.35	5%	12.18	2.13	5.72	2.44
DULOXETINE	N06A5 SNRI ANTIDEPRESSANTS	Yes	60	30 mg 28 caps	23.79	5%	5.93	0.98	6.05	1.39
MIANSERIN	N06A9 ANTIDEPRESSANTS ALL OTH	Yes	60	10 mg 30 tabs	13.37	3%	5.33	1.29	4.14	0.75

Note: XR—extended release. DDD—Defined Daily Dose.

**Table 3:** Interrupted time series analysis of drug consumption.

	Consumption		
Predictors	Estimates	CI	*p*
(Intercept)	36,656,603	34,342,014–38,971,192	<0.001
Period	398,673	248,797.8–548,548.2	<0.001
COVID fixed	137,746	−3,306,424–3,581,916	0.938
COVID variable	−47,282	−315,355–220,791.8	0.732
Observations	46		
R2/R2 adjusted	0.768/0.752		
F Statistic (df = 3; 42)	46.466		<0.001

**Table 4:** Regression results on the impact of COVID-19 on number of sick leave notes issued for diagnoses of anxiety and depression.

	Model											
	F32—Depressive Episode			F33—Recurrent Depressive Episode			F41—Other Anxiety Disorder			F43—Reaction to Severe Stress and Adjustment Disorder		
Predictors	Estimates	CI	*p*	Estimates	CI	*p*	Estimates	CI	*p*	Estimates	CI	*p*
(Intercept)	15,588.43	14,370.79–16,806.08	<0.001	9070.65	8286.10–9855.21	<0.001	11,804.04	10,276.14–13,331.94	<0.001	26,640.07	24,242.09–29,038.04	<0.001
Period	74.63	−4.21–153.48	0.063	15.13	−35.67–65.94	0.55	97.43	−1.51–196.36	0.053	247.29	92.02–402.57	0.003
COVID fixed	5621.17	3567.29–7675.04	<0.001	3328.46	2005.10–4651.81	<0.001	11,139.07	8561.88–13,716.27	<0.001	17,555.04	13,510.23–21,599.85	<0.001
COVID variable	−268.42	−483.32–−53.52	0.016	−230.90	−369.36–−92.44	0.002	−667.22	−936.87–−397.57	<0.001	−1216.00	−1639.21–−792.79	<0.001
Observations	40			40			40			40		
R2/R2 adjusted	0.761/0.741			0.570/0.534			0.845/0.832			0.862/0.851		
F Statistic (df = 3; 36)	38.22		<0.001	15.894		<0.001	65.354		<0.001	75.132		<0.001

## Data Availability

All publicly available data sources are cited in the text. Nonpublic datasets used in the analysis are available upon request from the corresponding author.
